# Contributions and Concerns about the Use of Teledentistry in Clinical Orthodontics

**DOI:** 10.3290/j.ohpd.b2081389

**Published:** 2021-09-30

**Authors:** Deema Al-Shammery, Nasser Alqhtani, Abdulelah N. Alotaibi, Mohammed AlSharidah, Khalid AlShehri, Alwaleed AlShamrani

**Affiliations:** a Assistant Professor, Department of Preventive Dental Science, College of Dentistry, Riyadh Elm University, Saudi Arabia. Study concept and design, data acquisition and analysis/interpretation, wrote the manuscript, critically revised and gave final approval to the article.; b Assistant Professor, Department of Oral and Maxillofacial Surgery and Diagnostic Sciences, College of Dentistry, Prince Sattam Bin Abdulaziz University, Al-Kharj, Saudi Arabia. Data acquisition and analysis/interpretation, wrote the manuscript, critically revised and gave final approval to the article.; c Dental Student, College of Dentistry, Riyadh Elm University, Saudi Arabia. Data acquisition and analysis/interpretation, wrote the manuscript, critically revised and gave final approval to the article.; d Dental Student, College of Dentistry, Riyadh Elm University, Saudi Arabia. Data acquisition and analysis/interpretation, wrote the manuscript, critically revised and gave final approval to the article.; e Dental Student, College of Dentistry, Riyadh Elm University, Saudi Arabia. Wrote the manuscript, critically revised and gave final approval to the article.; f Dental Student, College of Dentistry, Riyadh Elm University, Saudi Arabia. Wrote the manuscript, critically revised and gave final approval to the article.

**Keywords:** benefits, clinical, COVID, limitations, orthodontics, teledentistry, telemedicine

## Abstract

**Purpose:**

Teledentistry uses computer-based technology to render remote healthcare-related therapy and/or consultation. The purpose of this study was to review the contributions and concerns about the use of teledentistry in clinical orthodontics.

**Materials and Methods:**

The focused question was “What are the benefits and limitations of the use of teledentistry in clinical orthodontics?” PubMed/Medline, Scopus, Embase, Google-Scholar and ISI Web of knowledge databases were searched up to and including February 2021 using the following key words: 1. teledentistry; 2. teleorthodontics; 3. ethics; 4. orthodontics; 5. scope. The inclusion criteria were: (a) clinical studies; (b) case reports; and (c) case series. Studies on animal models, in vitro and/or ex vivo studies, letters, commentaries, and narrative and systematic reviews were not included in the search. The design of the study was tailored to recapitulate the relevant information.

**Results:**

Four clinical studies fulfilled the eligibility criteria and were processed for data extraction. All studies had been performed after obtaining informed consent from the participants. Three studies reported that teledentistry was useful in clinical orthodontics. In one study, a clear conclusion could not be drawn regarding the benefits of teledentistry in clinical orthodontics. Two out of the four studies did not obtain prior approval from an institutional review board or ethics committee. Three studies did not report any measures that were undertaken to safeguard the electronic transfer of patient-related health information.

**Conclusions:**

Teledentistry is a useful tool for initial patient assessments; however, it is not a reliable alternative for in-office clinical orthodontic practice.

Traditionally, healthcare-related services are offered during and/or after personal interaction between the healthcare providers and patients.^27^ According to the United States Department of Health and Human Services, vast oral health disparities exist across the country, which points towards the need to develop new modes of healthcare treatment that can improve access to care among different populations.^40^ Access to healthcare is often challenging for elderly patients and those living in rural areas.^41^ Teledentistry uses computer-based technology to provide remote healthcare treatment and/or consultation.^27,44^ It uses digital technology to facilitate the exchange of health records such as images (e.g. radiographs, CT scans), photographs, results of laboratory-acquired healthcare information and medications/prescriptions between healthcare providers and/or related institutions.^12^ Teledentistry has been successfully used in various disciplines of the oral and maxillofacial sciences;^27^ and has been reported to be similar to comparable to in-person oral screening.^2^ According to Duka et al,^14^ diagnostic evaluation of impacted teeth using teledentistry is as effective as real-time patient assessment. Moreover, in the field of dndodontics, teledentistry can be used for identification of root canal openings and periapical lesions in the anterior dentition.^8,24^ Nevertheless, teledentistry cannot be considered a reliable replacement for clinical in-person oro-dental evaluation. AlShaya et al^3^ tested the reliability of mobile-phone teledentistry in the diagnosis of caries among children. The authors observed that the reliability of teledentistry in terms of caries diagnosis was greater in the primary than in the permanent dentition. The authors concluded that without radiographs, teledentistry has limited accuracy in diagnosing caries.^3^

In a clinical orthodontic setting, diagnosis and treatment planning is dependent on an in-person patient evaluation, which encompasses clinical dento-skeletal evaluation, photographs of the facial profile, and digital or plaster models of the dentition. Since fixed orthodontic treatment requires adjustments in the dimensions of appliances, multiple follow-up visits to the operator/orthodontist and stringent oral hygiene maintenance are generally considered a pre-requisite.^29,32^ According to Khan and Omar,^27^ application of teledentistry in orthodontic practice enables individuals residing in rural or remote locations to obtain consultations and diagnosis of their dentoskeletal and cranio-facial anomalies. From the patient’s perspective, this strategy may also have cost-related benefits, particularly for individuals residing in remote locations, by minimising in-person visits to orthodontists.^6,22,31^ These results reflect that teledentistry can successfully be used for diagnosis and treatment planning of dento-skeletal malocclusions. Nevertheless, erratic visits to oral healthcare providers, including orthodontists, may negatively influence patient compliance with routine oral hygiene maintenance and stringent implementation of the planned orthodontic therapeutic protocol.^9,43^ From the authors’ point of view, a lack of routine dental health follow-up may compromise the outcome of the planned orthodontic treatment. Additionally, fallacies such as direct-to-consumer (DTC) orthodontics,^38^ which are devoid of routine professional supervision, trigger therapeutic complications and grave ethical issues.^41^ With emphasis on the current global COVID-19 pandemic, Giudice et al^18^ reported that teledentistry facilitates patient monitoring and simultaneously limits direct human contact, thereby minimising the chances of spread of this viral infection. However, Telles-Araujo et al^42^ suggested that teledentistry is not a reliable alternative for face-to-face patient visits, and whenever needed, every possible effort should be made to facilitate in-person patient examination.

It is hypothesised that teledentistry is a useful strategy for treatment planning and evaluation of patients undergoing orthodontic treatment. The purpose of this study was to review the contributions to and concerns about the use of teledentistry in clinical orthodontics.

## Materials and Methods

### Focus Question

The focus question was “What are the benefits and limitations of the use of teledentistry in clinical orthodontics?”

### Study Eligibility Protocol

The inclusion criteria were: (a) clinical studies; (b) case reports; and (c) case series. Studies on animal models, in vitro and/or ex vivo studies, letters, commentaries and narrative and systematic reviews were not included.

### Databases

Indexed databases (ISI Web of Knowledge, Embase, PubMed/Medline, OVID, and Google Scholar) were searched up to and including February 2021 without time and language limits. Boolean operators (AND / OR) were applied to the literature search using the following key indexing terms: Benefits; Limitations; Orthodontics; Clinical; Teledentistry; Telemedicine; COVID. Alhtough the present study is a narrative review, the authors adopted the Preferred Reporting Items for Systematic Reviews and Meta-Analyses (PRISMA) guidelines strategy.^36^ This was mainly done to identify and report the relevant data according to an organised and scientific approach.

## Results and Discussion

### Studies Related to the Benefits of Teledentistry in Clinical Orthodontics

**Fig 1:**
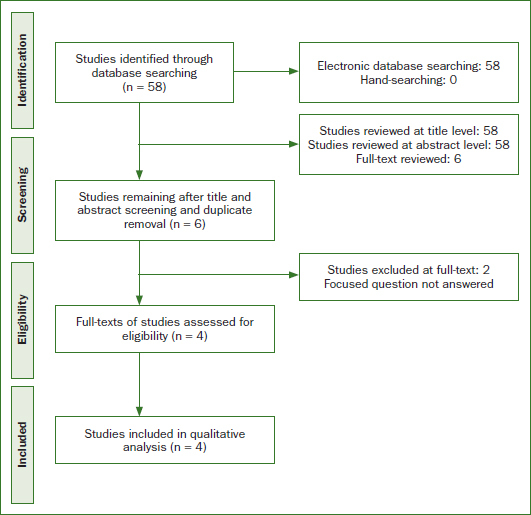
Study flowchart based on PRISMA guidelines.

Following an exhaustive literature search, 3 clinical studies^6,35,37^ that reported the benefits of teledentistry in clinical orthodontics were identified (Fig 1). In one study,^37^ the distance between maxillary canines and molars during maxillary expansion using plastic models (group 1) and dental monitoring software (group 2) were evaluated. The authors reported no statistically significant difference in the assessed parameters between groups.^37^ Berndt et al^6^ assessed patients who underwent consultation by a general-dentist for orthodontic treatment via teledentistry and compared the same treatment protocol provided by postgraduate residents in clinical orthodontics. Those authors^6^ reported that the treatment outcomes were comparable with both approaches. According to Mandall et al,^35^ a prior teledentistry-based orthodontic consultation facilitated referrals to orthodontists for future treatment.

### Studies with Inconclusive Outcomes

The literature search yielded one clinical study by Dunbar et al^15^ in which no definitive conclusion could be reached with reference to the values of teledentistry in clinical orthodontic practice.

### Prior Approval from Institutional Review Boards

All four studies^6,15,35,37^ had been performed after attaining informed consent from the participants. Nevertheless, the protocols of only 2 of the studies^15,37^ were authorised by the Institutional Review Board (IRB). Three of the studies^6,15,37^ did not report the measures adopted for the protection of patient-related data during electronic exchange.

### Possible Limitations of Teledentistry in Clinical Orthodontics

#### Identification and management of factors affecting orthodontic tooth movement

Identification of oral diseases, such as caries, endodontic infections and periodontitis, are a prerequisite for initiation of orthodontic therapy.^4,21,33^ Similarly, habits (e.g. smoking) and systemic conditions (e.g. diabetes mellitus, increased body mass index and mental disorders) may jeopardise the outcome of orthodontic treatment.^1,11,28,39^ There is abundant evidence to support the fact that medications including but not limited to bisphosphonates, thyroxine supplementation and non-steroidal anti-inflammatory drugs can affect the rate of orthodontically-induced tooth movement.^7,17,20,30,45^ This suggests that patient selection is an important factor that influences the overall success of orthodontically-induced tooth movement. It is proposed that patients using medications (such as those mentioned above) should be treated under direct operator supervision. It is further suggested that if smokers and patients with systemic diseases are solely treated using teledentistry, then it may be challenging to achieve the expected orthodontic results.

#### Written informed consent

In all clinical dental and orthodontic practice and related research, the importance of apriori informed consent cannot be overlooked. In addition, prior IRB approval for original research studies is mandatory to protect patients and participants and ensure the quality of the research projects.^34^ An astounding finding in the present review is that out of the 4 studies^6,15,35,37^ which described the potential application of teledentistry in clinical orthodontics, apriori IRB approval was missing in two^6,35^ of them. Therefore, it is suggested that outcomes shown in Table 1 are probably biased and hence a cautious interpretation of the results is recommended.

**Table 1:** Characteristics of included studies

Authors	Patients	Groups	Results	Informed consent given?	IRB approval?	Was tele-orthodontics useful?
Berndt et al^6^	126 patients	1: Patients received interceptive OT by a GD using teleorthodontics (n = 30) 2: Patients received interceptive OT by postgraduate residents under direct orthodontic faculty supervision (n = 96)	No statistically significant difference in the outcome of OT.	Yes	Not reported	Yes
Dunbar et al^15^	27 patients	1: Clinical examination-based orthodontic screening 2: Orthodontic screening based on hard copy or digital records without clinical examination	Intra- and inter-observer agreement varied among groups.	Yes	Yes	Unclear
Mandall et al^35^	327 patients from 15 dental practices	1: Referral to an orthodontist after teleorthodontics-based consultation 2: Direct referral to an orthodontist without prior teleorthodontic-based consultation	Inappropriate referrals were statistically significantly higher in group 2 than group 1.	Yes	Not reported	Yes
Moylan et al^37^	12 patients	1: Assessment of I/C and I/M widths using a dental monitoring software with smartphone 2: Assessment of I/C and I/M widths on plaster models	No statistically significant difference.	Yes	Yes	Yes

GD: general dentist; OT: orthodontic treatment; IRB: institutional review board; I/C: inter-canine; I/M: inter-molar.

#### Observation of patient compliance and tooth movement

Stringent yet routine oral hygiene maintenance plays an important role in the success of orthodontic treatment.^23^ It is well established that an increased plaque index and periodontal probing depth are risk factors for periodontal soft tissue inflammation and marginal bone loss around teeth.^25^ In a systematic review and meta-analysis, Huang et al^23^ quantitatively and qualitatively assessed studies with reference to the methods for improving motivation of oral hygiene in patients undergoing fixed orthodontic treatment. In that systematic review,^23^ 12 studies of moderate quality were assessed; the results showed that motivational efforts (in terms of oral hygiene maintenance) played a role in the overall success of fixed orthodontic treatment. Those authors^23^ concluded that orthodontists should put extra effort into encouraging their patients to practice routine oral hygiene maintenance. The present authors speculate that routine in-person dental visits to orthodontists and dental hygienists is a more reliable method to monitor the oral hygiene status of patients undergoing orthodontic treatment in contrast to sharing photos or videos via teledentistry. In addition, routine face-to-face dental visits may compel patients to regularly follow oral hygiene maintenance procedures. Another advantage of in-office visits over teledentistry is that complications such as oral mucosal lacerations (caused by factors such as broken orthodontic wires and brackets), caries, gingival inflammation and white-spot lesions can be instantly recognised and treated accordingly without compromising the duration and effectiveness of orthodontic treatment. From the authors’ point of view, such goals cannot be achieved via teledentistry.

The use of clear aligner therapy (CAT) is a modernisation in clinical orthodontics primarily because of its aesthetic advantages and relative “invisibility” compared with conventional fixed orthodontic appliances. However, factors such as addition and removal of attachments and interdental enamel reduction contribute towards the overall success of CAT.^10,19^ Thus, without direct supervision by an orthodontist or general dentist certified in CAT, the above-mentioned procedures cannot be done. The present authors suggest that because of such major limitations of teledentistry in clinical orthodontics, individuals opting to receive ‘do-it-yourself orthodontics’ should be cautioned.^31^ It is also important to mention that do-it-yourself orthodontics has been seriously criticised by other authors.^26^

#### Safeguarding the electronic transfer of patient-related health information

A concern associated with the digital transfer of patient records is breach of patients’ personal and health-related information.^13^ Over 20 years ago, the Health Insurance Portability and Accountability Act (HIPAA) was introduced to safeguard the exchange of patient-related health information between health professionals.^5^ It is notable that most of the studies^6,15,37^ which assessed the potential role of teledentistry in orthodontics did not undertake any steps (such as those detailed in HIPAA) to protect the electronic transfer of patient records. It has been recommended that all teledentistry-related efforts should provide sufficient security to meet the HIPAA standards.^16^ From the present authors’ perspective, these measures should be scrutinised by an IRB prior to study approval and enrollment of participants.

## Conclusion

Teledentistry is a useful tool in initial patient assessments; however, it is not a reliable alternative for in-office clinical orthodontic practice.

## References

[ref1] Al-Shammery D, Michelogiannakis D, Rossouw E, Romanos GE, Javed F (2019). Influence of psychological stress exposure on orthodontic therapy: A comprehensive review. J Investig Clin Dent.

[ref2] Alabdullah JH, Daniel SJ (2018). A Systematic Review on the Validity of Teledentistry. Telemed J E Health.

[ref3] AlShaya MS, Assery MK, Pani SC (2020). Reliability of mobile phone teledentistry in dental diagnosis and treatment planning in mixed dentition. J Telemed Telecare.

[ref4] Baeshen HA, Rangmar S, Kjellberg H, Birkhed D (2019). Dental caries and risk factors in Swedish adolescents about to start orthodontic treatment with fixed appliances. J Contemp Dent Pract.

[ref5] Banks DL (2006). The Health Insurance Portability and Accountability Act: does it live up to the promise?. J Med Syst.

[ref6] Berndt J, Leone P, King G (2008). Using teledentistry to provide interceptive orthodontic services to disadvantaged children. Am J Orthod Dentofacial Orthop.

[ref7] Berry S, Javed F, Rossouw PE, Barmak AB, Kalogirou EM, Michelogiannakis D (2020). Influence of thyroxine supplementation on orthodontically induced tooth movement and/or inflammatory root resorption: A systematic review. Orthod Craniofac Res.

[ref8] Brullmann D, Schmidtmann I, Warzecha K, d’Hoedt B (2011). Recognition of root canal orifices at a distance –a preliminary study of teledentistry. J Telemed Telecare.

[ref9] Charavet C, Le Gall M, Albert A, Bruwier A, Leroy S (2019). Patient compliance and orthodontic treatment efficacy of Planas functional appliances with TheraMon microsensors. Angle Orthod.

[ref10] Comba B, Parrini S, Rossini G, Castroflorio T, Deregibus A (2017). A Three-dimensional finite element analysis of upper-canine distalization with clear aligners, composite attachments, and class II elastics. J Clin Orthod.

[ref11] Consolaro A (2017). Obesity and orthodontic treatment: is there any direct relationship?. Dent Press J Orthod.

[ref12] Daniel SJ, Kumar S (2014). Teledentistry: a key component in access to care. J Evid Based Dent Pract.

[ref13] Dolezel D, McLeod A (2019). Managing security risk: modeling the root causes of data breaches. Health Care Manag (Frederick).

[ref14] Duka M, Mihailovic B, Miladinovic M, Jankovic A, Vujicic B (2009). [Evaluation of telemedicine systems for impacted third molars diagnosis]. Vojnosanit Pregl.

[ref15] Dunbar AC, Bearn D, McIntyre G (2014). The influence of using digital diagnostic information on orthodontic treatment planning –a pilot study. J Healthc Eng.

[ref16] Fricton J, Chen H (2009). Using teledentistry to improve access to dental care for the underserved. Dent Clin North Am.

[ref17] Friedrich RE, Scheuer HA, Höltje W The effect of bisphosphonate medication on orthodontics and orthognathic surgery in patients with osteogenesis imperfecta.

[ref18] Giudice A, Barone S, Muraca D, Averta F, Diodati F, Antonelli A (2020). Can teledentistry improve the monitoring of patients during the Covid-19 dissemination? A descriptive pilot study. Int J Environ Res Public Health.

[ref19] Gomez JP, Pena FM, Martinez V, Giraldo DC, Cardona CI (2015). Initial force systems during bodily tooth movement with plastic aligners and composite attachments: A three-dimensional finite element analysis. Angle Orthod.

[ref20] Gupta M, Kandula S, Laxmikanth SM, Vyavahare SS, Reddy SB, Ramachandra CS (2014). Controlling pain during orthodontic fixed appliance therapy with non-steroidal anti-inflammatory drugs (NSAID): a randomized, double-blinded, placebo-controlled study. J Orofac Orthop.

[ref21] Hamilton RS, Gutmann JL (1999). Endodontic-orthodontic relationships: a review of integrated treatment planning challenges. Int Endod J.

[ref22] Hansa I, Semaan SJ, Vaid NR, Ferguson DJ (2018). Remote monitoring and “Tele-orthodontics”: Concept, scope and applications. Semin Orthod.

[ref23] Huang J, Yao Y, Jiang J, Li C (2018). Effects of motivational methods on oral hygiene of orthodontic patients: A systematic review and meta-analysis. Medicine (Baltimore).

[ref24] Jampani ND, Nutalapati R, Dontula BS, Boyapati R (2011). Applications of teledentistry: A literature review and update. J Int Soc Prev Community Dent.

[ref25] Javed F, Näsström K, Benchimol D, Altamash M, Klinge B, Engström PE (2007). Comparison of periodontal and socioeconomic status between subjects with type 2 diabetes mellitus and non-diabetic controls. J Periodontol.

[ref26] Kannan S, Gowri S, Tyagi V, Kohli S, Jain R, Kapil P (2015). Direct-to-physician and direct-to-consumer advertising: Time to have stringent regulations. Int J Risk Saf Med.

[ref27] Khan SA, Omar H (2013). Teledentistry in practice: literature review. Telemed J E Health.

[ref28] Kirschneck C, Proff P, Maurer M, Reicheneder C, Römer P (2015). Orthodontic forces add to nicotine-induced loss of periodontal bone : An in vivo and in vitro study. J Orofac Orthop.

[ref29] Klaus K, Stark P, Serbesis TSP, Pancherz H, Ruf S (2017). Excellent versus unacceptable orthodontic results: influencing factors. Eur J Orthod.

[ref30] Krasny M, Zadurska M, Cessak G, Fiedor P (2013). Analysis of effect of non-steroidal anti-inflammatory drugs on teeth and oral tissues during orthodontic treatment. Report based on literature review. Acta Pol Pharm.

[ref31] Kravitz ND, Burris B, Butler D, Dabney CW (2016). Teledentistry, do-it-yourself orthodontics, and remote treatment monitoring. J Clin Orthod.

[ref32] Li X, Xu ZR, Tang N, Ye C, Zhu XL, Zhou T (2016). Effect of intervention using a messaging app on compliance and duration of treatment in orthodontic patients. Clin Oral Investig.

[ref33] Lu H, Tang H, Zhou T, Kang N (2018). Assessment of the periodontal health status in patients undergoing orthodontic treatment with fixed appliances and Invisalign system: A meta-analysis. Medicine (Baltimore).

[ref34] Mallia P (2018). WASP (Write a Scientific Paper): Ethics approval for a research study (1). Early Hum Dev.

[ref35] Mandall NA, O’Brien KD, Brady J, Worthington HV, Harvey L (2005). Teledentistry for screening new patient orthodontic referrals. Part 1: A randomised controlled trial. Br Dent J.

[ref36] Moher D, Liberati A, Tetzlaff J, Altman DG (2009). Preferred reporting items for systematic reviews and meta-analyses: the PRISMA statement. PLoS Med.

[ref37] Moylan HB, Carrico CK, Lindauer SJ, Tufekci E (2019). Accuracy of a smartphone-based orthodontic treatment-monitoring application: A pilot study. Angle Orthod.

[ref38] Okuda BC, Tabbaa S, Edmonds M, Toubouti Y, Saltaji H (2021). Direct to consumer orthodontics: Exploring patient demographic trends and preferences. Am J Orthod Dentofacial Orthop.

[ref39] Saloom HF, Boustan R, Seehra J, Papageorgiou SN, Carpenter GH, Cobourne MT (2020). The impact of obesity on orthodontic treatment outcome in adolescents: a prospective clinical cohort study. Eur J Orthod.

[ref40] Simon L, Friedland B (2016). Interstate practice of dental teleradiology in the United States: the effect of licensing requirements on oral and maxillofacial radiologists’ practice patterns. Telemed J E Health.

[ref41] Squires T, Michelogiannakis D, Rossouw PE, Javed F (2021). An evidence-based review of the scope and potential ethical concerns of teleorthodontics. J Dent Educ.

[ref42] Telles-Araujo GT, Caminha RDG, Kallás MS, Santos P (2020). Teledentistry support in COVID-19 oral care. Clinics (Sao Paulo).

[ref43] Wu TY, Kuang SH, Wu CH (2009). Factors associated with the stability of mini-implants for orthodontic anchorage: a study of 414 samples in Taiwan. J Oral Maxillofac Surg.

[ref44] Zimlichman E (2005). Telemedicine: why the delay?. Isr Med Assoc J.

[ref45] Zymperdikas VF, Yavropoulou MP, Kaklamanos EG, Papadopoulos MA (2020). Effects of systematic bisphosphonate use in patients under orthodontic treatment: a systematic review. Eur J Orthod.

